# Fine-tuning genomic and pedigree inbreeding rates in equine population with a deep and reliable stud book: the case of the Pura Raza Española horse

**DOI:** 10.1186/s40104-022-00781-5

**Published:** 2022-11-07

**Authors:** Davinia Isabel Perdomo-González, Nora Laseca, Sebastián Demyda-Peyrás, Mercedes Valera, Isabel Cervantes, Antonio Molina

**Affiliations:** 1 Departamento Agronomía, Escuela Técnica Superior de Ingeniería Agromómica, Universidad de Sevilla, Ctra Utrera Km 1, 41013 Sevilla, Spain; 2 Departamento de Genética, Universidad de Córdoba, Córdoba, Spain; 3 Facultad de Ciencias Veterinarias, Universidad Nacional de La Plata, La Plata, Argentina; 4 Consejo Nacional de Investigaciones Científicas y Técnicas (CONICET), La Plata, Argentina; 5 Departamento de Producción Animal, Facultad de Veterinaria, Universidad Complutense de Madrid, Madrid, Spain

**Keywords:** Genomic inbreeding, Horses, Pedigree inbreeding

## Abstract

**Background:**

Estimating inbreeding, which is omnipresent and inevitable in livestock populations, is a primary goal for management and animal breeding especially for those interested in mitigating the negative consequences of inbreeding. Inbreeding coefficients have been historically estimated by using pedigree information; however, over the last decade, genome-base inbreeding coefficients have come to the forefront in this field. The Pura Raza Española (PRE) horse is an autochthonous Spanish horse breed which has been recognised since 1912. The total PRE population (344,718 horses) was used to estimate Classical (*F*), Ballou’s ancestral, Kalinowski’s ancestral, Kalinowski’s new and the ancestral history coefficient values. In addition, genotypic data from a selected population of 805 PRE individuals was used to determine the individual inbreeding coefficient using SNP-by-SNP-based techniques (methods of moments -*F*_HOM_-, the diagonal elements of the genomic -*F*_G_-, and hybrid matrixes -*F*_H_-) and ROH measures (*F*_RZ_). The analyse of both pedigree and genomic based inbreeding coefficients in a large and robust population such as the PRE horse, with proven parenteral information for the last 40 years and a high degree of completeness (over 90% for the last 70 years) will allow us to understand PRE genetic variability better and the correlations between the estimations will give the data greater reliability.

**Results:**

The mean values of the pedigree-based inbreeding coefficients ranged from 0.01 (*F* for the last 3 generations -*F*3-) to 0.44 (ancestral history coefficient) and the mean values of genomic-based inbreeding coefficients varied from 0.05 (*F*_RZ_ for three generations, *F*_H_ and *F*_HOM_) to 0.11 (*F*_RZ_ for nine generations). Significant correlations were also found between pedigree and genomic inbreeding values, which ranged between 0.58 (*F*3 with *F*_HOM_) and 0.79 (*F* with *F*_RZ_). In addition, the correlations between *F*_RZ_ estimated for the last 20 generations and the pedigree-based inbreeding highlight the fact that fewer generations of genomic data are required when comparing total inbreeding values, and the opposite when ancient values are calculated.

**Conclusions:**

Ultimately, our results show that it is still useful to work with a deep and reliable pedigree in pedigree-based genetic studies with very large effective population sizes. Obtaining a satisfactory parameter will always be desirable, but the approximation obtained with a robust pedigree will allow us to work more efficiently and economically than with massive genotyping.

## Background

In populations of a genetically finite size, the mating of closely-related individuals is inevitable even in large populations, resulting in inbred offspring. Inbreeding, with the accompanying increase in the genome homozygosity, results in a reduction of genetic diversity and is often related with a phenomenon known as inbreeding depression, which is the reduction in performance per unit increase in inbreeding coefficient [1–3]. The magnitude of inbreeding depression often poses a considerable threat to the survival of inbred populations [4]. Although conservation geneticists and those responsible for genetic improvement plans are constantly looking for efficient strategies to overcome the negative consequences of inbreeding [5], as optimal contribution selection strategies [6], such strategies can not be implemented systematically in all populations. For this reason, it is important to obtain accurate estimates of inbreeding in order to permit a better management of the animal populations under selection.

Historically, in animal populations, inbreeding has been calculated from pedigree information and extended literature exists to estimate different pedigree-base inbreeding coefficients such as classical inbreeding [7, 8], ancestral inbreeding [9, 10], partial inbreeding coefficients [11, 12] and the ancestral history coefficient [13]. Nevertheless, as the cost of genotyping falls, genomic inbreeding coefficients can also be easily obtained. Genomic inbreeding coefficients are expected to be more accurate than pedigree-based coefficients because they do not depend on the quality and completeness of the pedigree. In addition, genomic inbreeding coefficients measure real homozygosity while pedigree-based inbreeding coefficients make average estimations.

While pedigree-based inbreeding coefficients use deterministic or stochastic methods to distinguish recent from ancestral inbreeding, genomic inbreeding attempts to detect the proportion of the genome covered by homozygous regions (runs of homozygosis, ROH) of a certain length, following the theory proposed by Fisher [14]. Knowledge about the proportion of recent and ancestral inbreeding is especially relevant in order to identify if an individual’s ancestor has been also subjected to inbreeding. Animals with inbred ancestors are less susceptible to inbreeding depression than individuals with non-inbred ancestors, due to the purging effects associated with ancestral inbreeding. Therefore, looking for signs of potential purging provides a better mirroring of the genetic load than simply measuring classical inbreeding [15].

In the same way as pedigree-based inbreeding coefficients, genomic inbreeding values can also be estimated with different metrics. The simplest SNP-based methods include the methods of moments technique [16] and inbreeding derived from the diagonal of the genomic-relationship matrix [17, 18]. More recently, the methodologies based on the estimation of ROH have become the state-of-art procedures. Among them, observational approaches, in which ROH are determined by a moving window of fixed size which scans each chromosome to determine the presence of a certain number of consecutive homozygous markers (implemented in PLINK [16]) are the most commonly used in livestock studies [19]. However, their reliability is lower in comparison with those based on the use of hidden Markov models [20], which estimates the probabilities of identity by descent at each marker of an individual [21]. Nowadays, it is still open to debate which methodology provides the most reliable and accurate inbreeding estimations, even more so when the number of studies that compare them with the pedigree records is still extremely low [22] and when different thresholds and constraint could be applied within each genomic estimating methodology [19].

The Pura Raza Española (PRE) is one of the oldest European horse breeds and the best known in the Iberian Peninsula, with over 250,000 active individuals. In addition, PRE horses are currently distributed in over 60 countries but managed by a single association, the Real Asociación Nacional de Criadores de Caballos de Pura Raza Española (ANCCE) which carries out breeding as a meta-population, a term which refers to a group of populations with some possible gene flow among them [23]. The PRE studbook was created in 1912, and from that moment, animals were only registered if at least two complete generations were known. Next, the studbook was completely closed and registration of new PRE horses was restricted to animals with parents already registered. Later, in the early 1980’s, paternity controls were carried out in PRE horses using different molecular tools, such as blood grouping, serum biochemical polymorphism and DNA microsatellites [24–26]. As a result, the PRE population has over 40 years of proven parental information, as well as having a large and deep pedigree with a high degree of completeness [27], which has remained over 90% for the last 70 years (the last 7 generational intervals). Since its studbook creation, both ANCCE and PRE breeders have been working to maintain the genetic variability of breeding. In fact, inbreeding increased exponentially up to 8.5% in the 1980s, and then decreased to the current figure of 7.5% due to measures to control inbreeding carried out in recent years.

In horses, populational genomic studies are still scarce in comparison with other livestock species. Nowadays, the availability of array-based SNP genotyping (from 65 K to 670 K markers per individual) in the species is increasing, allowing for the development of genomic tools and studies aimed at characterizing populations [28–30] and determining the genetic basis of traits [31], among others. However, despite the fact that the number has recently grown [32–34], ROH-based studies in large cohorts and populations of horses are still very scarce [35].

The aim of this paper was to analyse the evolution of 8 pedigree and 4 different genomic-based inbreeding estimations in a large cohort of nearly 300,000 horses with a large and robust pedigree. In addition, we determined the correlations among all the estimations (pedigree and genomic based) using partial correlations in order to establish the reliability of different inbreeding estimations in horses.

## Methods

### Dataset

In this study, the genealogical information of all individuals registered in the PRE horse studbook was analysed, in which 97.97% (337,712 horses) have at least 3 complete generations. The total PRE population is composed of 344,718 horses (168,301 males and 176,417 females), born from the end of the nineteenth century to 2020. The mean of equivalent generations was 9.46 and the average numbers of full and maximal generations traced were 5.66 and 17.17, respectively. Animals with no known parents in the pedigree data were considered as founders and assumed to be unrelated.

### Pedigree-based inbreeding coefficients

Pedigree-based inbreeding coefficients were computed using Endog [36] and GRain [13] programmes. The classical inbreeding coefficient (*F*) according to Wright [7] is defined as the probability that the two alleles at any locus in an individual are identical by descent (IBD). In addition, depending on the number of generations taken into account, inbreeding coefficients at the 3^rd^, 6^th^ and 9^th^ generations can be computed (*F*3, *F*6 and *F*9, respectively). Otherwise, ancestral inbreeding coefficients were calculated following the approaches by Ballou [9] (*F*a_Bal) and Kalinowski et al. [10] (*F*a_Kal). While *F*a_Bal is defined as the probability that any allele in an individual has been autozygous (IBD) in previous generations at least once, *F*a_Kal is defined as the probability that any allele in an individual is currently IBD and has been IBD in previous generations at least once. At the same time, the Kalinowski approach allows us to split *F* into two parts: alleles which have undergone inbreeding in the past (*F*a_Kal) and alleles IBD which have done so for the first time (*F*new_Kal). On the other hand, partial inbreeding coefficients according to Lacy et al. [11, 12] (*F*_ij_) examine whether alleles contributing to inbreeding have been distributed uniformly across founder genomes or from specific founders. Finally, the ancestral history coefficient, developed by Baumung et al. [13], has been defined as the number that tells us how many times, during the pedigree segregation, a randomly-taken allele has had IBD status. The idea behind distinguish recent and ancestral inbreeding coefficients is that alleles which have experienced inbreeding more often in the past are less likely to be deleterious than alleles which have undergone IBD less often.

### SNP genotyping

Genotypic data from 805 PRE individuals was analysed, whose selection was based on a low average relatedness from 365 studs. To do this, blood samples of each individual were obtained by jugular venepuncture using sterile tubes with EDTA. Next, DNA was obtained from 200 μL of whole blood using commercial kits following the manufacturer’s instructions.

The genotypes of 670,776 SNPs markers were determined for each individual using the HD Axiom™ Equine SNP Genotyping Array (Thermofisher, Madrid, Spain). The raw data (.CEL files) were first analysed following the “*best genotyping practices*” workflow in the Axiom Analysis Suite 5.0 software [37]. All the samples passed the genotyping quality threshold (dish quality check ≥ 0.82 and plate call rate ≥ 0.97). However, only 540,294 SNPs markers (located in 31 chromosomes) showing a high-quality genotyping rate (SNP call rate > 95% and Fisher’s Linear Discriminant parameter > 3.6) were kept for inbreeding analysis. No minor allele frequency or linkage disequilibrium filtering was performed, following the latest ROH estimation guidelines [19].

### Genomic-based inbreeding coefficients

Four different approaches were employed to determine the individual inbreeding coefficient using genomic data. First, we carried out a multistep methodology based on a hidden Markov model (HMM) framework developed by Druet and Gautier [21], which take into account the allele frequency of each SNP, its genetic position, and the genotyping error rate. All the ROH per individual were determined using a 7-class model (K = 6, 12, 18, 36, 72, 144, and 144), with a *mixing coefficient* = 0.01, and a *genotyping error probability* = 0.001, implemented in the RZooROH R package [38]. Therearter, the individual inbreeding value, *F*_RZ_, was estimated as the relationship between the length of the genome covered by ROH (defined as continuous homozygous stretches with a minimum length of one megabase in the genome) and the total genome length of the genome fragments, as proposed by McQuillan et al. [39] using the *summaryRuns* function of *DetectRUNS* R package [40]. In this estimation, only ROH longer than 1 Mb were taking into account to avoid detecting ROH IBD segments [19].

Inbreeding was also calculated as the individual autozygosity (diagonal) of the genomic (*F*_G_) and hybrid (*F*_H_) matrixes proposed by VanRaden [17] and Martini et al. [18], respectively. The matrixes were constructed using only the genomic data (***G***) and a combination of genomic data and pedigree records available (***H***) using the AGHMatrix R package [41]. Finally, the method-of-moments methodology was applied to estimate *F*_HOM_, the inbreeding coefficient as the difference between the observed and the expected homozygotes counts per individual. The calculations were made using the *--het* option in PLINK v1.90 [16].

In addition, genomic inbreeding value explained by matings occurred during the last 20 generations was determined following the theoretical approach proposed by Fisher [14] to analyse the theoretical increase of individual inbreeding across the last 20 generations. To do this, all the ROH fragments detected per individual using the RZooROH package were analysed, and 20 iterative runs were performed in which only ROH with a minimum length of 1/2 g Morgans (where g is the number of generations theoretically elapsed since the inbreeding event) were retained in the inbreeding calculation. This produced 19 additional inbreeding values (*F*_RZG2_ to *F*_RZG20_) per individual.

### Statistical analysis

Mean values for the different pedigree-based inbreeding coefficient estimations for the total PRE population and the genotyped individuals were calculated. The total PRE population was divided according to each generational interval (each generational interval comprising 10 years, [42]) to analyse the evolution of founders and common ancestors and the partial inbreeding coefficients (*F*_ij_) transmitted by them. While the effective number of founders (fe) defines the number of equally contributing founders that would be expected to produce the same genetic diversity as in the total population, the effective number of ancestors (fa) refers to the minimum number of ancestors, not necessarily founders, which account for the complete genetic diversity, as in the total population.

In addition, for every trio of inbreeding coefficients, *x*, *y* and *z*, the three first-order partial correlation coefficients were computed to estimate the correlation between each pair of parameters with the third estimated variables fixed (partial correlation). The partial correlation coefficient between *x* and *y* given *z* indicates the strength of the linear relationship between *x* and *y,* that is, independent of, and uncorrelated with, *z*. The comparison with the ordinary (or unconditional or zero-order) correlation coefficient, allows us to determine whether the association between the two inbreeding coefficients has been sharply reduced after eliminating the effect of the third inbreeding coefficient. For every trio of inbreeding coefficients, in order to obtain the tolerance level (*ε*) to be used as the local threshold for determining significant associations, the mean ratio of partial to direct correlation was calculated according to Reverter and Chan [43]:

$$\mathcal{E}=\frac{1}{3}\left(\frac{r_{xy,z}}{r_{xy}}+\frac{r_{xz,y}}{r_{xz}}+\frac{r_{yz,x}}{r_{yz}}\right)$$

with *r*_*xy,z*_, *r*_*xz,y*_, and *r*_*yz,x*_ the three partial correlations, and *r*_*𝑥𝑦*_, *r*_*𝑥z*_ and *r*_*yz*_ the ordinary correlations.

A correlation between inbreeding coefficients *x* and *y* is discarded if:

$$\left|{r}_{xy}\right|\le \left|\mathcal{E}\ {r}_{xz}\right|\kern0.5em and\kern0.5em \left|{r}_{xy}\right|\le \left|\mathcal{E}\ {r}_{yz}\right|$$

Otherwise, the association is defined as significant.

This procedure was extended to all the coefficient parameters different from *x* and *y* to determine which correlations exceeded the estimated thresholds and could be considered non-spurious relationships [44]. The first-order partial correlation coefficients together with the thresholds for determining significant associations were calculated with the software *PCIT* package in R [45].

### Data handling

Management of the pedigree record and molecular dataset was performed entirely in the R environment using the following packages: *dplyr* [46], *tibble* and *tidyr* from *tidyverse* [47], and *data.table* [48]. Data visualization was conducted in *ggplot2* [49].

## Results

### Population structure

The number of individuals born in each generational interval (Table 1) has increased since the year 1950. The average *F* increased to 8.4% in 1980–1989, then started to decrease, a trend which continues to date (7.28%). The number of founders decreased until the generational interval of 1990–1999, when there was a slight increase, followed by a decrease in the next generations. Similar results can be seen for ancestors and equivalent founders. Nevertheless, while the number of founders, equivalent founders and ancestors have remained relatively constant over the history, the number of effective founders and effective ancestors have decreased to almost half. The numbers of effective founders and ancestors have oscillated from 58 to 34 and from 33 to 19, respectively, over the last 70 years, while the fe/fa ratio for each generational interval has varied between 1.66 at the 1980–1989 generational interval to 1.78 at the 2010–2019 generational interval. Finally, the average common ancestors (those in both, maternal and paternal linage) of individuals in each generational interval has increased from 18.29 in the 1950–1959 interval (transmitting an average *F*_ij_ of 0.73%) to 242.40 in the present day (transmitting an average *F*_ij_ of 0.03%).

**Table 1:** Inbreeding, founders, ancestors, and common ancestors of Pura Raza Española horse population by generational intervals

GI	N	*F*, %	Funders	NEF	Ancestors	fe	fa	fe/fa	MCA [Min, Max]	*F*_ij_, %
1950–1959	1818	6.87	410	330	350	58	33	1.75	18.29 [1, 63]	0.73
1960–1969	2357	6.73	407	323	348	44	26	1.69	28.45 [1, 132]	0.26
1970–1979	6046	7.48	387	309	344	34	20	1.70	49 [1, 173]	0.17
1980–1989	17,091	8.40	394	317	351	30	18	1.66	81.83 [1, 248]	0.12
1990–1999	53,102	8.32	424	337	394	29	17	1.70	124.76 [1, 336]	0.08
2000–2009	150,129	7.45	411	324	383	32	19	1.68	183.36 [26, 521]	0.05
2010–2019	109,062	7.28	339	315	379	34	19	1.78	242.40 [34, 771]	0.03

*GI* Generational interval, *N* number of individuals born, *F* mean classical inbreeding, *NEF* number of equivalent founders, *fe *effective number of founders, *fa* effective number of ancestors, *fe/fa* ratio between fe and fa, *MCA* mean common ancestors, *Min* minimum number of common ancestors, *Max* maximum number of common ancestors, *F*_ij_ mean partial inbreeding coefficient

**Fig. 1:**
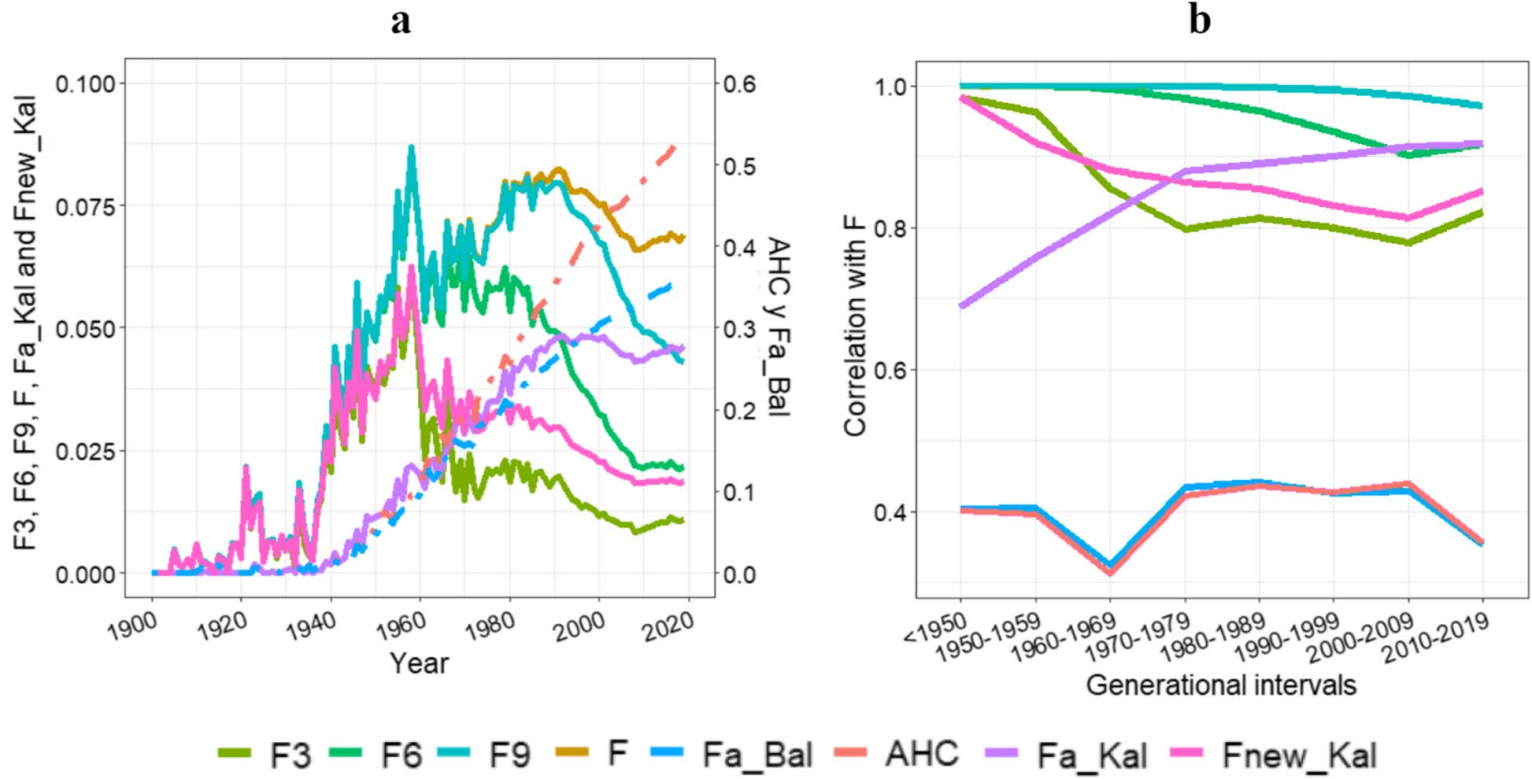
Pedigree-based inbreeding coefficients evolution and correlations with the classical inbreeding coefficient by generational intervals. Evolution of pedigree-based inbreeding coefficients of the total PRE population (**a**); evolution of correlations between the different inbreeding coefficients and the classical inbreeding coefficient by generational intervals (**b**); Inbreeding coefficients at 3^rd^, 6^th^, and 9^th^ generations (*F*3, *F*6 and *F*9, respectively); classical inbreeding coefficient (*F*); ancestral Ballou inbreeding coefficient (*F*a_Bal); ancestral Kalinowski inbreeding coefficient (*F*a_Kal); new Kalinowski inbreeding coefficient (*F*new_Kal) and ancestral history coefficient (AHC)

The evolution of the different pedigree-based inbreeding coefficients over the years can be seen in Fig. 1a. All the pedigree-based inbreeding coefficients started to increase around 1940. While AHC and *F*a_Bal increased exponentially to 0.541 and 0.367 by 2019, the other coefficients increased more rapidly around the 1960s but then has started to decrease in recent years, with some values appearing constant. In 2019, *F*3, *F*6 and *F*new_Kal showed similar values, 0.011, 0.023 and 0.022, respectively, while *F*9 and *F*a_Kal also showed similar values between them, 0.047 and 0.051, respectively, and finally, *F* was 0.074.

The correlations between *F* and the other pedigree-based inbreeding coefficients (Fig. 1b) reveal differences over the generational intervals. The correlation values for *F*a_Bal and AHC seem to be the lowest, at around 0.4, while those for the other coefficients were over 0.8 for most of the intervals. *F*9 and *F*6 had the most constant coefficients always close to 1 while the *F*a_Kal correlation values increased from 0.69 to 0.91. However, *F*new_Kal and *F*3 correlation values have fallen over the last 50 years, at nearly 0.8.

### Pedigree-based inbreeding estimations

The different pedigree-based inbreeding coefficients mean values and Pearson’s correlations between them for the total PRE population and genotyped individuals can be seen at Table 2. The mean values ranged between 0.01 (*F*3) and 0.08 (*F*) for most pedigree-based inbreeding coefficients, except for *F*a_Bal (0.31) and AHC (0.44). Most of the Pearson’s correlations between pedigree-based inbreeding coefficients were significant, positive and with moderate to high value. Similar correlation values can be seen between inbreeding coefficients for the total population and for genotyped individuals. The highest positive significant values were found between *F* and *F*9 (0.98 and 0.99 for the total population and genotyped individuals, respectively) and between *F*a_Bal and AHC (0.98 and 0.99 for total population and genotyped individuals, respectively). The lowest positive significant values were found between *F*a_Bal and *F* (0,45) and between *F*a_bal and *F*9 (0,36), both for genotyped animals. There were no negative and significant correlations between pedigree-based inbreeding coefficients.

**Table 2:** Pearson’s correlations between pedigree-based inbreeding coefficients for genotyped individuals and total population

	Mean	*F*3	*F*6	*F*9	*F*	*F*a_Bal	AHC	*F*a_Kal	*F*new_Kal
*F*3	0.01		0.93^a^	0.81^a^	0.79^a^	−0.07	− 0.10	0.45	0.96^a^
*F*6	0.03	0.89^a^		0.93^a^	0.90^a^	0.05	0.01	0.62	0.95^a^
*F*9	0.06	0.81^a^	0.95^a^		0.99^a^	0.36^a^	0.33	0.85^a^	0.84^a^
*F*	0.07	0.79^a^	0.91^a^	0.98^a^		0.45^a^	0.42	0.89^a^	0.81^a^
*F*a_Bal	0.31	−0.01	0.02	0.22	0.36		0.99^a^	0.76^a^	−0.10
AHC	0.44	−0.02	0.01	0.22	0.36	0.98^a^		0.74^a^	−0.13
*F*a_Kal	0.04	0.52	0.68	0.85^a^	0.90^a^	0.60^a^	0.63^a^		0.46
*F*new_Kal	0.02	0.92^a^	0.94^a^	0.87^a^	0.84^a^	−0.07	−0.08	0.53	

Mean values are for different pedigree-based inbreeding coefficient estimations of the total PRE population. Over the diagonal: Pearson’s correlations between pedigree-based inbreeding coefficient for genotyped individuals; under the diagonal: Pearson’s correlations between pedigree-based inbreeding coefficient for total PRE population.^a^Significant according to Watson-Haigh et al. [45]. Standard mean errors were lower than 0.0005. Inbreeding coefficients at 3^rd^, 6^th^, and 9^th^ generations (*F*3, *F*6 and *F*9, respectively), classical inbreeding coefficient (*F*), ancestral Ballou inbreeding coefficient (*F*a_Bal), ancestral history coefficient (AHC), ancestral Kalinowski inbreeding coefficient (*F*a_Kal) and new Kalinowski inbreeding coefficient (*F*new_Kal)

### Genomic-based inbreeding estimations

The average results of the 4 different genomic inbreeding coefficients, Pearson’s correlations within the genomic-based inbreeding coefficients and the Pearson’s correlations between the genomic and pedigree inbreeding coefficients are shown at Table 3. The average values show clear differences based on the methodology employed. Within the ROH-based methodology (*F*_RZ_), the average values were 0.05, 0.09 and 0.11 for *F*_RZ3_, *F*_RZ6_, *F*_RZ9_, respectively, while for the total generations (*F*_RZ_), it was 0.17. In comparison, the matrixial and method-of-moments based methods showed low and very similar values (0.05, 0.06 and 0.05 in *F*_H_, *F*_G_ and *F*_HOM_, respectively).

**Table 3:** Pearson’s correlations within genomic and between genomic and pedigree-based inbreeding coefficient estimations for genotyped animals

		Genomic inbreeding						Genealogical inbreeding							
	Mean	*F*_RZ3_	*F*_RZ6_	*F*_RZ9_	*F*_G_	*F*_H_	*F*_HOM_	*F*3	*F*6	*F*9	*F*	*F*a_Bal	AHC	*F*a_Kal	*F*new_Kal
*F*_RZ_	0.17	0.85^a^	0.94^a^	0.97^a^	0.41	0.26	0.98^a^	0.59^a^	0.68^a^	0.78^a^	0.79^a^	0.4	0.38	0.72^a^	0.61^a^
*F*_RZ3_	0.05		0.94^a^	0.91^a^	0.67^a^	0.52	0.84^a^	0.75^a^	0.77^a^	0.75^a^	0.75^a^	0.15	0.13	0.55	0.75^a^
*F*_RZ6_	0.09			0.99^a^	0.56^a^	0.39	0.93^a^	0.68^a^	0.74^a^	0.78^a^	0.78^a^	0.28	0.25	0.65^a^	0.70^a^
*F*_RZ9_	0.11				0.51	0.34	0.95^a^	0.64^a^	0.72^a^	0.78^a^	0.78^a^	0.32	0.29	0.68^a^	0.66^a^
*F*_G_	0.06					0.83^a^	0.42	0.74^a^	0.68^a^	0.45	0.46	−0.31	−0.32	0.13	0.73^a^
*F*_H_	0.05						0.26	0.79^a^	0.71^a^	0.49	0.42	−0.43	−0.46	0.03	0.77^a^
*F*_HOM_	0.05							0.58^a^	0.67^a^	0.76^a^	0.78^a^	0.39	0.37	0.71^a^	0.60^a^

Mean values are for different genomic-based inbreeding coefficient estimations of the PRE genotyped animals; Pearson correlations within genomic inbreeding coefficients (left part) and between genomic and pedigree-based inbreeding coefficient estimations (right part).^a^Significant according to Watson-Haigh et al. [45]. Standard means errors were lower than 0.0005. ZooROH approach genomic inbreeding coefficient for all generations and 3, 6, and 9 generations (*F*_RZ_, *F*_RZ3_, *F*_RZ6_, *F*_RZ9_, respectively), genomic inbreeding based on the autozygosity (the matrix diagonal) using genomic data (*F*_*G*_, matrix ***G*** according to VanRaden [17]), and genomic and pedigree data (*F*_H_, matrix ***H*** according to Martini et al. [18]), inbreeding coefficient using Plink (*F*_HOM_), inbreeding coefficients at 3^rd^, 6^th^, and 9^th^ generations (*F*3, *F*6 and *F*9, respectively), classical inbreeding coefficient (*F*), ancestral Ballou inbreeding coefficient (*F*a_Bal), ancestral history coefficient (AHC), ancestral Kalinowski inbreeding coefficient (*F*a_Kal) and new Kalinowski inbreeding coefficient (*F*new_Kal)

The Pearson’s correlations within the genomic-based inbreeding coefficients are mostly, positive and of a high magnitude. *F*_RZ_, *F*_RZ3_, *F*_RZ6_ and *F*_RZ9_ showed a significant, high correlation between each other and with *F*_HOM_, ranging between 0.84 (*F*_RZ3_-*F*_HOM_) and 0.99 (*F*_RZ6_-*F*_RZ9_). On the other hand, while *F*_G_ showed high and significant correlation values with *F*_RZ3_ (0.67) and *F*_RZ6_ (0.56), the other correlations between *F*_G_ and *F*_H_ and the other of the genomic estimations were lower and not significant. Interestingly, the correlation between both matrix-based methodologies (*F*_H_ and *F*_G_) was high and significant (0.83).

### Correlations between pedigree and genomic based inbreeding estimations

The Pearson’s correlations between the genomic and pedigree-based inbreeding coefficients (right part of Table 3) were mostly significant, positive and of a high magnitude, ranging between 0.58 (*F*3-*F*_HOM_) and 0.79 (*F*-*F*_RZ_). The classical pedigree-based estimation (*F*) showed the best fit with the non-matrixial genomic estimations among all the pedigree-based *F* values, including 0.79 for *F*-*F*_RZ_ and 0.78 for *F*-*F*_HOM,_ *F*-*F*_RZ6G_ and *F*-*F*_RZ9G_. On the contrary, *F*_G_ and *F*_H_ showed significant correlations with pedigree data only when the information of 3 or 6 generations was employed in the estimations. The pedigree-based AHC and *F*a_Bal showed low, non-significant correlations with all the pedigree and genomic estimations, showing negative values with *F*_H_ and *F*_G_. Finally, both Kalinowski estimations showed high and significant correlations with the rest of the genomic and pedigree based values. However, the ancestral Kalinowski estimation showed low correlations with both matrixial estimators (*F*_G_ and *F*_H_).

**Fig. 2:**
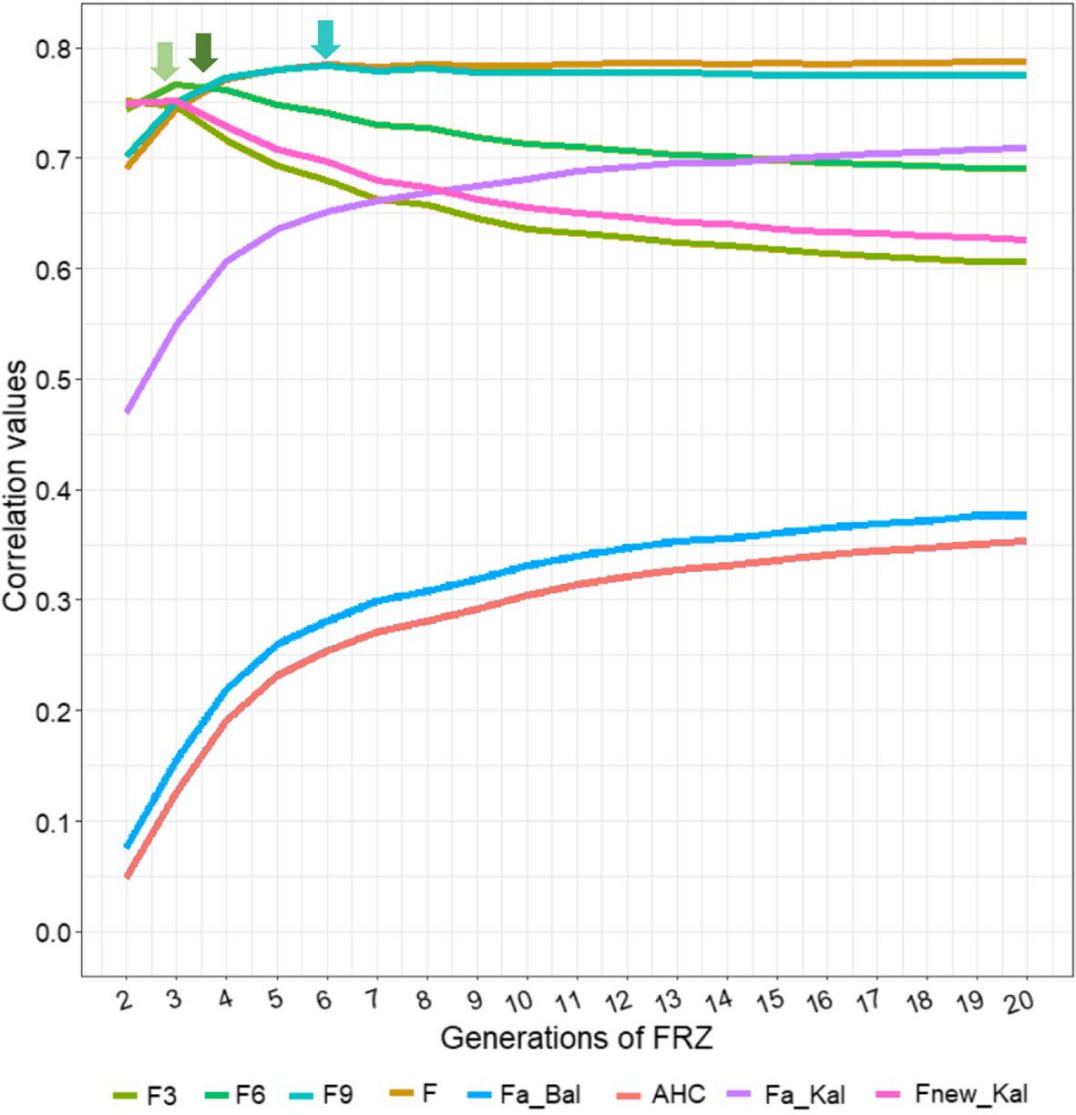
Pearson’s correlations between pedigree-based and *F*_RZ_ inbreeding for the last 20 generations. The ZooROH approach genomic inbreeding coefficient (*F*_RZ_) was determined, which had elapsed since the hypothetical inbred mating in the PRE population; Inbreeding coefficients at 3^rd^, 6^th^, and 9^th^ generations (*F*3, *F*6 and *F*9, respectively); classical inbreeding coefficient (*F*); ancestral Ballou inbreeding coefficient (*F*a_Bal); ancestral Kalinowski inbreeding coefficient (*F*a_Kal); new Kalinowski inbreeding coefficient (*F*new_Kal) and ancestral history coefficient (AHC). The narrows depict the best correlations obtained with *F*3, *F*6 and *F*9

The correlations between the genomic inbreeding values estimated for the last 20 generations (from generation two, *F*_RZ_-2G, to generation 20, *F*_RZ_-20G) and the pedigree-based inbreeding estimations are shown in Fig. 2. Partial correlation values with *F*3, *F*6, *F*9 and *F* showed a similar pattern, in which correlations increased up to a certain point and then started to decrease with the increase of generations considered. Interestingly, the best correlation for *F*3 was observed using genomic data from only 3 generations (*F*_RZ_-3G). Similarly, the best correlation for *F*6 was observed using 4 generations of genomic data (*F*_RZ_-4G) and the best correlation for *F*9 was observed using 6 generations of genomic data (*F*_RZ_-6G). In contrast, *F*a_Kal showed an increasing correlation value across the generations, with the best correlation at *F*_RZ_-20G, whereas *F*a_Bal and AHC showed the lowest correlation values with *F*_RZ_ of all the analyses performed.

## Discussion

Inbreeding estimations based on pedigree information can help us to understand the population structure and trends in inbreeding in each breed, and has been historically used to manage populations and control inbreeding depression within populations. Nevertheless, it is not enough to know the population inbreeding level, as it does not exactly reflect the real degree of genome homozygosis, because inbreeding is not directly related to inbreeding depression, since natural and artificial selection over time may have meant that deleterious recessive alleles are purged or advantageous recessive alleles are fixed in a population [50–53]. Here, the distinction between ancient and recent inbreeding is also a highly relevant factor, since inbreeding arising from a distant common ancestor should have less effect on fitness than inbreeding from recent common ancestors [54]. For this reason, different methods of pedigree-based inbreeding estimations have been developed. However, there have been few studies in which they have been compared [44, 55–58] and none have analyzed all the different methods together in a large population with a deep, well-establishe, complete pedigree. In these sense, the PRE is an exceptional case in which to analyse the different methodologies of pedigree-based inbreeding estimations and compare them with genomic-based parameters where the 98.7% of the total PRE population is inbred (with an *F* value higher than 0) [27].

The PRE population census has undergone uneven progress during the generational interval analysed (Table 1). The number of horses born in latest generational interval is over one hundred thousand times that in the 1950s. The fe/fa ratio (wich describe the unbalanced representation of the founder contributions) for each generational interval found in this study has undergone major changes in the genetic management of the population and demonstrated that different genetic bottlenecks have taken place along the history of the PRE horse. The bottleneck phenomenon has also been seen in other horse breeds, such as the Old Kladruber horse [59], the Spanish Arab Horse [60] and the Campolina horse population [61] with an fe/fa ratio of 5.40, 2.03, and 1.51, respectively, although all of these have a much smaller clearly census. On the other hand, the increase in the average number of common ancestors and the decrease in the mean partial inbreeding coefficient that those common ancestors transmit to their descendants agrees with the lastest studies of the genetic structure of the PRE population [27] and reflects a problem which is increasingly worrying of breeders, the selection of unrelated animals for breeding, instead of leading to an increase in genetic diversity in the PRE population, results in a loss.

Table 2 shows the mean values of the pedigree-based inbreeding coefficient of the total PRE population and, as expected, when different pedigree-based inbreeding coefficients are compared, the average values increase from recent to ancient inbreeding estimates. The total population average *F*a_Bal value (0.31) was seven times higher than the total population average *F*a_Kal value (0.04). By definition, *F*a_Bal is a value which tells us which individuals or populations possess fewer detrimental genes. It therefore follows that, the higher the value of *F*a_Bal, the lower the probability of having detrimental genes [57]. As can be seen in Fig. 1, average *F*a_Bal values have been increasing from the 1940s and we can safely say that on average, the PRE population is prone to limiting incidents of inbreeding depression as the generations progress. Studies analysing the differences between *F*a_Bal and *F*a_Kal have also shown higher values for *F*a_Bal as in the case of the German Anger and the Red-and-White cattle breeds [57], and a crossbred rabbits population [44]. Nevertheless, Suwanlee et al. [62] found that Ballou’s formula overestimate the real proportion of alleles within a genome which has undergone inbreeding by stochastic simulations of different settings for population size and initial allele frequencies, even though the overestimation seems to be more pronounced in small populations. Similar behaviour can be observed in Fig. 1 for AHC for each generational interval, the average value has increased to the current value of 0.51. Our results show that, according to the total population average value (Table 2), an allele at random gene of a random individual have been IBD 0.44 times. In this sense, it has been shown in a population of Australian Thoroughbred horses [63], with a smaller pedigree (257,249 horses), but with an *F* value (0.139) and a mean equivalent generation (24.6) higher than the PRE population, and an AHC value of 1.973, that this coefficient best captures the effectiveness of selective breeding practices in increasing the frequency of favourable alleles and the purging of highly and mildly deleterious alleles. In the populations of German Anger and the Red-and-White cattle breeds studied [57], AHC and *F*a_Bal values were also the highest inbreeding coefficient values and had very similar values between them, with an AHC value of 3.94 and a *F*a_Bal value of 3.69 for German Anger and an AHC value of 1.49 and a *F*a_Bal value of 1.39 for the Red-and-White breeds, while in the crossbred rabbit population [44], the AHC value was three times higher than *F*a_Bal, 2.72 and 0.85, respectively.

Pearson’s correlations between pedigree-based inbreeding coefficients (Table 2) were similar for genotyped individuals to that of the total population, which reveals the representativeness of the sample selected for genotyping. While positive and significant correlations were detected between classical inbreeding coefficients and classical inbreeding coefficients in different generations (*F*3, *F*6 and *F*9), no significant correlations were found between them with *F*a_Bal and AHC. On the other hand, high positive significant correlations were found here between *F*a_Kal with *F*a_Bal (0.60 for total population and 0.76 genotyped individuals) and AHC (0.63 and 0.74), being lower than those obtained in Thoroughbred horses [63], 0.90 *F*a_Kal-*F*a_Bal, and 0.85 *F*a_Kal-AHC, but similar to those in a combined pedigree of the German Anger and the Red-and-White cattle breeds populations [57], 0.63 *F*a_Kal-*F*a_Bal and 0.65 *F*a_Kal-AHC. Nevertheless, both Mc Parland et al. [64] in an Irish Holstein-Fresian cattle population and Schäler et al. [55] in the Angler saddleback pig population reported weak correlation values between them, while Rodríguez-Ramilo et al. [44] reported no significant correlations in crossbred rabbits. At the same time, high, positive significant correlations were found here between *F*a_Kal with *F*9 (0.85) and *F* (0.90), which is in line with both Rodríguez-Ramilo et al. [44] and Schäler et al. [55]. The correlation between *F*a_Bal and AHC (0.98) was also positive, strong and significant in Rodríguez-Ramilo et al. [44], Addo et al. [57] and Todd et al. [63]. Finally, *F*new_Kal had high, positive correlations with *F*3 (0.92), *F*6 (0.94), *F*9 (0.87), and *F* (0.84) but no significant correlations with ancestral coefficients (*F*a_Bal, AHC, and *F*a_Kal). Ultimately, the high inbreeding coefficients on PRE population are more related to ancestral inbreeding than to recent inbreeding, which seem to be under control, and thus implies being less prone to inbreeding depression due to purging.

Although the classical inbreeding coefficient has often been considered as the best measure of population inbreeding, it may be an unrealistic measure of individual IBD. The variability between real levels of autozygosity and probability-based estimations can be due to recombination (with a stochastic nature) and a change on allele frequencies due to selection, in addition to the fact that pedigree-based inbreeding coefficients depend on the completeness of studbook and the reliability of the documentation [65]. Many of the problems evidenced in pedigree-based inbreeding can be overcome by using different methods based on analysis of the genomic data, which has been stated as a more precise estimate of IBD [65, 66]. Despite that studies analysing the correlation between pedigree and genomic-based inbreeding coefficients have shown strong correlations between genomic and pedigree-based inbreeding coefficients in human [39], cattle [67–69], rabbit [44] or pig populations [55], the data avaible in horse is still scarce.

An unresolved question is still how to determine the most reliable methodology to estimate genomic-based inbreeding values among the current alternatives available since comparison of their results can be contradictory due to diverse factors [22]. For instance, recent studies have reported that ROH-based *F* measures (*F*_RZ_ and *F*_ROH_) are more powerful in detecting inbreeding depression than SNP-by-SNP-based F measures (*F*_G_, *F*_H_ and *F*_HOM_) [65, 70], but even more when using information from medium-density arrays since the accuracy of the laters strongly relies on the number of markers per individual [71]. Moreover, Caballero et al. [72] indicated that the use of *F*_G_ (named *F*^I^ in that study), and *F*_HOM_ (to a lesser extent), was not advised to estimate inbreeding depression; as well as Villanueva et al. [73] reported that matrixes have proven to be very effective in increasing the accuracy of genomic predictions, however, they do not always provide a useful measure of inbreeding. Similarly, Ceballos et al. [20] found that methodologies based on statistical models that estimate probabilities of IBD at each marker such as hidden Markov models were more reliable and robust since they take into account more factors to estimate inbreeding, such as marker allele frequencies, genetic distances, genotyping error rates and the sequences of observed genotypes. In addition, hidden Markov model-based methodologies allow to provide a better fit for individual genetic data and to refine the genomic partitioning of inbreeding into stretches of IBD segments from possibly different ancestral origins [21]. In this sence, a recent comprehensive meta-analysis [22] which compared the performance of genomic methodologies employed in this study (*F*_RZ_, *F*_HOM_, *F*_G_ and *F*_H_) suggested that hidden Markov model-based *F* estimations (such as *F*_RZ_) seems to be the more reliable. Same results were obtained in this study, when they were compared with the classical inbreeding coefficient *F*, but also with several different pedigree-based inbreeding estimators. However, the same authors mentioned that the size and genetic structure of the population evaluated must be taken into account to consider one methodology better than the other, suggesting that the best fitting genomic estimates for a population of a relatively small size (< 1000) are those based on ROH (*F*_RZ_), while those based on SNP by SNP analysis (*F*_HOM_, *F*_G_ and *F*_H_) are better for large populations (10,000) [74]. This hypothesis agrees with our results.

The correlations among different genomics *F* measures showed that matrixial methods (*F*_H_, and to a lesser extent *F*_G_) are less related and showed lower average values in comparison with the rest of the genomic measures. On the contrary, *F*_HOM_ and *F*_RZ_ showed the highest correlation (0.98), in agreement with the reported by Solé et al. [75] in which the correlations between hidden Markov model-based estimates and measures based on homozygosity and ROH were extremely high (*F*_HOM_, *r* = 0.95 and *F*_ROH_, *r* = 0.95, respectively). Moreover, they found that the correlations between the model based on the matrix proposed by VanRaden [17] and *F*_HOM_ and *F*_RZ_ were lower, agreeing with our results. Similar results were provided by Caballero et al. [72], which reported the lowest correlation between the matrix-based method (*F*^I^) and hidden Markov model based methodologies. In addition, we obtained a correlation of 0.42 between *F*_G_ and *F*_HOM_ higher than obtained by Villanueva et al. [73], whose values were 0.25 and 0.28 for two independent datasets. Our results support the previous data suggesting that the estimations of inbreeding values obtained from matrixial methods should be used with caution, since both methods tends to underestimate the existence of ancestral inbreeding, and therefore, the *F* value. In a recent study, Meyermans et al. [19] used the RZooRoH (*F*_RZ_) model based approach to validate their study of runs of homozygosity using PLINK. They obtained a high Pearson correlation of individual *F*_ROH_ between PLINK and RZooRoH (*F*_RZ_) (0.89–0.99) which was similar to our previous results (0.95) [76]. Therefore, in agreement with above results, we believe it is a robust methodology for estimating genomic inbreeding.

At the present day, genomic estimates of *F* are considered more reliable and accurate than the pedigree-based for two main reasons. First, pedigree based estimations depend on the quality of the pedigree data available, which sometimes is scarce and/or not entirely reliable [71]. But also, pedigree-based estimations assume an equal distribution of the “inbreeding” across the entire genome as well as a proportional passage of IBD alleles from the common ancestor, which has been proven as unrealistic in several species [33, 69, 71, 77]. However, the validation of this fact in a livestock population with a deep and robust pedigree is still scarce. In our study, the genealogical estimations were made by using the Pura Raza Española studbook pedigree, which deepness and reliability is largely proven.

Our results showed high correlations between classical pedigree-based estimation (*F*) and genomic estimations *F*_RZ_ (0.79) and *F*_HOM_ (0.78) in comparison with recent studies in other breeds such as the Mangalarga Marchador horse (0.02) [33]. However, those differences are more likely to be produced by the fact that Mangalarga pedigree included only 4 equivalent generations, in comparison with the 9.46 (and 17.17 maximal generations in average) of the Pura Raza Española horses. This effect produced by the incompleteness of pedigree information has been previously pointed as a serious constrain for the estimation of the real value of inbreeding [78]. In this sense, Polak et al. [79] reported an increase in the correlation coefficient between pedigree-based *F* and *F*_ROH_ along with the increase of the number of generations registered in pedigree data, which was also observed by [33] which used a pedigree database with a median depth of 15 generations. Our results are in agreement with both reports, highlighting the importance of the pedigree robustness in the estimation of inbreeding. Similarly, two recent studies compared genomic-based with pedigree-based inbreeding coefficients in the Norwegian–Swedish Coldblooded trotter and the Polish Cold-Blooded horses [79, 80]. In the second one, authors reported a moderate correlation between pedigree (*F*_PED_) and genomic (*F*_ROH_) inbreeding estimations, being 0.56 the highest value. In the first [79], authors found similar correlations between genealogical *F* and the genomic coefficient estimated based on ROH (0.443), but even lower when genomic inbreeding was estimated using the diagonal of the genomic relationship matrix. This agrees with our results, as the highest correlations were between pedigree-based *F* coefficients and ROH-based *F* measures (*F*_RZ_) and the weakest between inbreeding coefficients based on the diagonal of the genomic (*F*_G_) and hybrid (*F*_H_) matrixes. However, it also need to be taking into account the differences in the number of individuals genotyped (805 in our case, 566 and 192 in their studies), as well the use of individuals from different generations, which is also required for an accurate estimation of *F* using pedigree values [22].

It was noteworthy that the inclusion of pedigree information in the ***G*** matrix (by estimating the hybrid matrix ***H***) improved the correlations of this methodology with pedigree-based methods but decreased the same correlations with genomic-based estimations. Despite the fact this is highly expectable, Martini et al. [18] demonstrated that the best fitting is highly dependant on the the correct estimation of τ and ω coefficients, and therefore, it should be adjusted very carefully.

Although most studies only correlated genomic inbreeding measures with the classical inbreeding coefficient (*F*), we have also made correlations between genomic estimates with ancestral inbreeding coefficients (*F*a_Bal and *F*a_Kal). *F*a_Bal was moderately correlated with *F*_RZ_ (0.4) and *F*_HOM_ (0.39). This was in accordance with Schäler et al. [55], whose found a correlation of 0.39 between *F*a_Bal and *F*_HOM_, and 0.49 between *F*a_Bal and *F*_ROH_ (ROH-based measure as *F*_RZ_). However, their results do not agree with our correlations between *F*a_Kal and *F*_HOM_ and *F*_RZ_. While our correlations were high and significant between *F*_HOM_ and *F*_RZ_ (0.71 and 0.72, respectively), their correlations with *F*a_Kal were low and non-significant (0.19 and 0, respectively). This can be explained not only by the lower genomic data analysed (from 76 individuals) but also by the population structure since Schäler et al. [55] analysed a population pedigree of 1273 individuals with an average *F* of 0.03, an average *F*a_Bal of 0.024 and an average *F*a_Kal of 0.09, which implies that recent inbreeding is more worrying than in the PRE population, whose recent inbreeding has been controlled. In addition, the results suggest that matrixial methods are less accurate in capturing the ancestral inbreeding than those based on more complex algorithms.

Nowadays, it is a common practice to determine recent and ancient inbreeding using the pedigree information of 3, 6, 9, or ROH fragments longer than 16.6, 8.3 and 5.5 Mb in pedigree and genomic-based estimations respectively. In our case, it was first employed a more refined approach by estimating the hypothetycall *F*_RZ_ value during the last 20 generations, according to the minnimun size of IBD fragments described by Fisher [14] (Fig. 2). Best correlations for *F*_3_, *F*_6_ and *F*new_Kal were observed using genomic data from 3, 4, and 20 generations (according the length of the ROH), demonstrating good fit. However, correlations decays in the three cases when shoerter ROH fragments were included, which is highly expectable since all of them better estimate the recent inbreeding. On the contrary, *F*_9_ and *F* increased the correlations rapidly with genomic values estimated using the first 6 generations, after which they become asymptotic in a value close to 0.78 despite the inclusion of more genomic information into the analysis. But also, this fits with the average pedigree information available on the PRE database (close to 9 generations on average), suggesting that the inclusion of additional genomic information associated to this value (ROH shorter that 5 Mb aproximately) is futile. However, it is also noteworthy that the differences observed among correlations before the lines start to decay (between 2^nd^ and 5^th^ generation in *F*_3_, *F*_6_ and *F*new_Kal) or become asymptotic (from 9^th^ to 20^th^ generation in *F* and *F*_9_) are extremely low (less than 2% in average), and therefore, we can state that the correlation between genomic and pedigree-based estimations follows accurately the model proposed by Fisher [14] 70 years ago.

On the contrary, both ancestral estimators (AHC and *F*a_Bal) as well the original *F*a_Kal showed an increased correlation with the ROH-based genomic estimations, which was more marked when the genomic information included was lower than 7 generations (ROH < 7.1 Mb, according to Fisher [14]), after which the inclusion of additional genomic information did not provided a great improvement. However, it is noteworthy that AHC and *F*a_Bal showed extremely lower correlations, in agreement with their aim in capturing the ancestral *F*, whereas *F*a_Kal showed much higher values which were even better (in terms of correlations) with the obtained *F*_3_ and *F*new_Kal after the 9^th^ generation and after the 15^th^ in *F*_6_. Interestingly, our results fits adequately with both, the original theory of Fisher and the later validation of Howringan [81], but in this case, based on the analysis of a real horse population worldwide bred as the Pura Raza Española horse.

Finally, Wang [82] suggested that pedigrees cannot be replaced completely by genomic data, because the pedigree estimations allow for the calculation of more complicated IBD coefficients for which the genomic estimations may have reduced capacity or limited power. Additionally, Todd et al. [63] suggested that the use of pedigree data allows inferences to be made for individuals from many generations ago, for whom biological samples might not be available for genotyping, particularly in horse.

## Conclusions

In conclusion, our results show that if the pedigree has sufficient depth and reliability (especially if it includes individuals from different generations), the estimates obtained with the classical parameters present an acceptable correlation and therefore continue to be the most useful for the reproductive management of populations, even in this genomic era. In addition, the high correlation between the classical *F* and Kalinowski’s *F* allows us to ensure that, in the current population, the possible founder effect is sufficiently diluted. In fact, the comparation of correlation between the *F*_RZ_ and the two previous pedigree-based estimates shows a slightly higher correlation of *F*_RZ_ with the classical *F*.

Obviously, it will also be preferable to obtain a real parameter instead of an estimated average, and the substantial incorporation of genomic information in livestock breeding programs gives us the opportunity to develop and implement new routines to manage populations at the genomic level. Nevertheless, the approximation obtained with a robust pedigree will allow us to work efficiently and more cheaply than with massive genotyping of the population, if the economic cost is a limitation. Obviously, in those populations in which there is a poor pedigree, the use of genomic information is the only valid way to obtain parameters for the genetic management of said population.

## Data Availability

The dataset supporting the results of this study was supplied by the National Association of Pura Raza Española Horse Breeders (ANCCE). The datasets generated and/or analysed during the current study are available from the corresponding author upon reasonable request.

## Abbreviations

AHC: Ancestral history coefficient
*F*: Wright classical inbreeding value
fa: Number of ancestors
*F*a_Bal: Ballou’s ancestral inbreeding
*F*a_Kal: Kalinowski’s ancestral inbreeding
fe: Number of founders
*F*_G_: Genomic inbreeding value by the diagonal elements of the genomic matrix
*F*_H_: Genomic inbreeding value by the diagonal elements of the hybrid matrix
*F*_HOM_: Genomic inbreeding value applying the method-of-moments methodology
*F*_i__j_: Partial inbreeding coefficients
*F*new_Kal: Kalinowski’s new inbreeding
*F*_RZ_: Genomic inbreeding value applying the RZooROH R package
*F*3: Classical inbreeding considering 3 generations
*F*6: Classical inbreeding considering 6 generations
*F*9: Classical inbreeding considering 9 generations
IBD: Identity by descent
ROH: Runs of homozygosity
